# Selection of patient-reported outcome measures (PROMs) and patient-reported experience measures (PREMs) for long-term follow up of patients diagnosed with lung or rectal cancer

**DOI:** 10.1186/s41687-026-01133-x

**Published:** 2026-07-08

**Authors:** Gina Linmans, Svin Deneckere, Charlotte Lens, Esther van Poel, Cindy De Gendt, Walter Sermeus, Dirk De Ridder

**Affiliations:** 1 Department of Public Health and Primary Care, Leuven Institute for Healthcare Policy, KU Leuven, Leuven, Belgium; 2 Flemish Institute for Quality of Care, Ghent, Belgium; 3 Ghent University, Ghent, Belgium; 4 Belgian Cancer Registry, Brussels, Belgium

**Keywords:** Patient reported outcome measurements, PROMs, Patient reported experience measurements, PREMs, Lung cancer, Rectal cancer

## Abstract

**Background:**

Despite their recognized value, the heterogeneous use of PROMs and PREMs prevents standardized data collection across hospitals, limiting their impact on both individual patient care and broader healthcare optimization. This study aims to identify and establish a standardized set of PROMs and PREMs for lung and rectal cancer to support uniform data collection and follow-up.

**Methodology:**

This multicenter study involved 10 Flemish hospitals focusing on lung and rectal cancer and applied a nine-phase methodology: (1) literature-based identification of PROMs and PREMs, (2) inventory of PROMs and PREMs currently used and proposed by the participating hospitals, (3) systematic assessment of PROM and PREM characteristics, (4) preliminary selection by the research team, (5) a Modified Delphi study with clinical experts, (6) an interview with methodologists, (7) patient focus groups, (8) a consensus meeting with clinical experts, and (9) finalization by the research group. A total of 13 lung cancer experts and 13 rectal cancer experts participated in the modified Delphi study. In addition, interviews were conducted with two methodologists, and five lung cancer patients and five rectal cancer patients, including both current and former patients, took part in the focus groups.

**Results:**

Consensus-based standardized sets of PROMs for lung and rectal cancer were selected. The final PROM sets consisted of the EQ-5D-5L (generic), EORTC QLQ-C30 and adapted PRO-CTCAE (cancer-specific), EORTC QLQ-LC29 (lung cancer-specific), and the EORTC QLQ-CR29, EORTC QLQ-LMC21 (1 item) and LARS (rectal cancer-specific), and an adapted version of the SF-HLQ for employment-related outcomes in both cancer populations. No PREM met the selection criteria.

**Conclusion:**

Based on a structured multi-phase approach integrating quantitative and qualitative methods with input from both patients and clinical experts, standardized PROM sets were selected for lung and rectal cancer. A specific PREM needs to be developed.

**Supplementary Information:**

The online version contains supplementary material available at https://doi.org/10.1186/s41687-026-01133-x.

## Background

With early diagnosis and better treatments, the number of cancer survivors is increasing worldwide. The World Health Organization (WHO) estimates that 53.5 million people are alive within five years of diagnosis; consequently, cancer is increasingly regarded as a chronic disease [1–3]. In Belgium, 77,344 people were diagnosed with invasive cancer in 2023, with lung and rectal cancer making up about 12.3% and 2.7% of cases [4, 5]. Currently, healthcare quality indicators mainly focus on care processes and survival, but they often miss key elements that matter most to patients, such as psychosocial well-being, symptom burden, and health-related quality of life [1]. Understanding patients’ perspectives is vital, as involving them as active partners and co-producers in care can improve outcomes [6]. The Triple Aim framework, which focuses on improving patient experience, population health, and cost efficiency, highlights that enhancing care quality and patients’ quality of life creates value. This concept has been extended in the Quadruple and Quintuple Aim to also include healthcare provider well-being and health equity, reinforcing the importance of patient-centered outcome measurement [7–9].

Patient-reported outcomes (PROs), reflecting patients’ health assessment, are increasingly vital for improving quality of care [1]. They are gathered via questionnaires called patient-reported outcome measures (PROMs), which assess physical health, mental health and daily functioning. Patient-reported experience measures (PREMs) assess the patients’ healthcare experiences, including satisfaction with care and information provided, accessibility, coordination, waiting times, and provider interactions [10]. PROMs can improve shared decision-making, patient-centered care, patient–provider communication, and help identify unmet needs, during treatment monitoring and follow-up [10, 11]. In addition, studies have shown that systematic PRO monitoring during cancer treatment can improve symptom control, quality of life, and even overall survival. In lung cancer specifically, PROMs are increasingly used in both clinical trials and routine care to monitor symptom burden, treatment toxicity, and functional outcomes [12, 13]. PREMs are mainly used to evaluate and improve healthcare delivery at an aggregated level. However, the benefits of patient-reported measures at the hospital level, such as quality improvement, benchmarking, collaborative learning, and public reporting, and at the policy level, such as healthcare evaluation and value-based care, are far less clear. International initiatives led by organizations such as the organization for Economic Co-operation and Development (OECD) advocate for the standardized use of PROMs and PREMs to support patient-centered care [14].

Despite their relevance, systematic use of PROMs and PREMs in daily practice remains limited. Although PROMs are increasingly used, particulary in lung cancer care [15, 16], implementation remains fragmented across hospitals, with limited standardization, patient involvement, and alignment with primary care [17–19]. Evidence from implementation studies in lung cancer shows that the benefit of PROMs depends not on their collection alone, but on their integration into structured care pathways with active follow-up, suggesting that effective implementation is key to achieving benefit [20, 21]. However, such structural implementation is not yet established in Flanders. In addition, PREMs are frequently collected using generic instruments that do not provide actionable insights at the level of specific departments or care pathways, such as oncology. This heterogeneity results in misalignment regarding the use and interpretation of PROMs and PREMs, both in individual care and in the broader healthcare process. Standardization of measurement is therefore essential to generate valid and comparable data for quality evaluation and improvement.

This study aims to select standardized minimum PROM and PREM sets for patients with lung and rectal cancer, with input from clinical experts and patients, given the high incidence and public health impact of both cancer types.

## Methods

### Phase 1: Literature-based identification of PROMs and PREMs

**Fig. 1:**
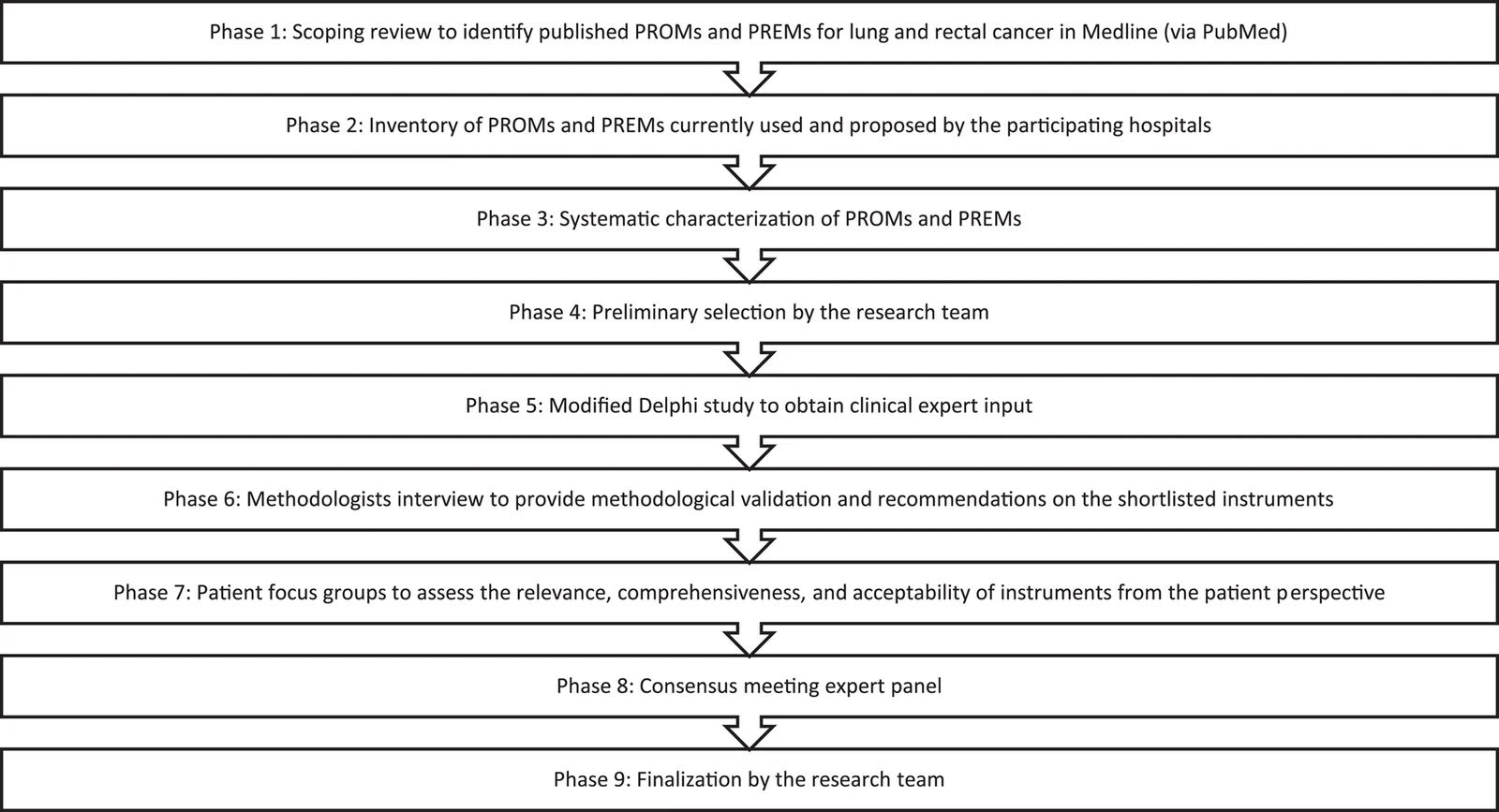
Chronological overview of the PROMs and PREMs selection process

Standardized minimal sets of PROMs and PREMs for lung and rectal cancer were selected using a structured multi-phase approach, as illustrated in Fig. 1. First, a scoping review was conducted. The search in Medline (via PubMed) on November 15, 2022, used the following terms: ‘Patient-Reported Outcome Measures’, ‘Patient-Reported Experience Measures’, ‘Rectal cancer’, ‘Lung cancer’, with MeSH terms, synonyms and wildcards (asterisks). The following filters were applied: published maximum 10 years ago, English, Dutch, abstract, full text, and human. Title screening was done by two independent researchers (GL and CL). Questionnaires reported in at least two abstracts were included, and categorized as generic, cancer-specific, lung cancer-specific, or rectal cancer-specific.

### Phase 2: Inventory of PROMs and PREMs currently used and proposed by the participating hospitals

Ten Flemish hospitals with a formal commitment to implementing PROMs and PREMs for lung and rectal cancer were asked to specify which PROMs and PREMs they currently use or consider relevant for inclusion in the project.

### Phase 3: Systematic assessment of PROM and PREM characteristics

An extensive online search was conducted for all identified PROMs and PREMs, including those retrieved from the literature and those submitted by the hospitals, to gather comprehensive information on nine characteristics: domains covered, length/items, response categories, normative data, costs, available languages, validity, reliability, and prognostic value. The search utilized scientific databases such as Medline and Google Scholar, as well as grey literature sources (e.g. organizational reports, PROM repositories such as the EORTC Quality of Life Group database, FACIT.org and the ePROVIDE platform, and official instrument manuals).

### Phase 4: Preliminary selection by the research team

To ensure feasibility of the modified Delphi study, the research team conducted a preliminary selection of PROMs and PREMs. The remaining questionnaires, together with the related information from the literature search (Phase 3), were subsequently provided to the expert panels.

### Phase 5: A modified Delphi study with clinical experts

Two modified Delphi studies [22], one for lung cancer and one for rectal cancer, were conducted with clinical experts (e.g., gastroenterologists, pulmonologists, oncologists, surgeons and specialized nurses) from the 10 participating hospitals. Information collected during the literature search (Phase 3) was summarized in a standardized format and provided to the experts. The Delphi evaluation focused on predefined measurement and implementation characteristics rather than detailed item-level content review. Therefore, full questionnaire texts were not systematically distributed. Experts primarily evaluated the instruments based on the standardized summaries provided by the research team. However, if experts wished to consult the full questionnaire text, they could either retrieve it independently or request it from the researcher (GL), after which it was provided when available.

Experts evaluated each instrument on validity, importance, feasibility, and usefulness using a 7-point Likert scale ranging from “strongly disagree” (1) to “strongly agree” (7). Providing scores on these criteria was optional; however, an overall score (1–7) for each instrument was mandatory. Instruments were categorized based on the proportion of experts assigning an overall score of six or seven: green (≥ 75% of experts, i.e. at least 10 out of 13), yellow (50–74%, i.e. 7–9 experts), and red (< 50%, i.e. ≤6 experts). The threshold of 75% was chosen to ensure a high level of agreement among researchers, with scores of six or seven on a seven-point scale reflecting instruments considered to be of high quality.

### Phase 6: An interview with methodologists

Two methodological experts were consulted to provide an independent perspective on the selection process and the preliminary set of questionnaires. They were provided with an overview of the study objective, the selection procedure, and the questionnaires identified up to that stage. Specifically, they were asked to reflect on the methodological approach and selection criteria, and to provide recommendations regarding the relevance and applicability of the questionnaires for the intended study context.

### Phase 7: Patient focus groups

To involve patients in selecting PROMs and PREMs, focus groups were conducted with participants recruited from the 10 participating hospitals and through patient associations affiliated with the Flemish Patients’ Platform. Eligibility criteria included current or previous treatment for lung or rectal cancer, age ≥ 18 years, sufficient language proficiency, and the ability to attend the focus group in person. Participants received study materials beforehand, including an explanation of PROMs and PREMs, the study purpose, their role in selecting appropriate instruments and full-text versions of the preselected questionnaires. Participants were asked to review the questionnaires in advance. During the focus groups, a semi-structured interview guide was used to explore the relevance, comprehensiveness, feasibility, perceived strengths and weaknesses, and burden of the questionnaires. Participants were also asked about their preferences between instruments, preferred scoring formats, administration mode (digital or paper), recall period, language availability, and whether questionnaire items should be mandatory. All participants provided informed consent. The focus groups were moderated by a representative of the Flemish Patients’ Platform, and participants were reimbursed for their participation. Each focus group lasted approximately two hours. Audio recordings were transcribed verbatim and analyzed using a thematic analysis approach combining deductive and inductive elements. Initial coding was guided by predefined research questions, while emerging themes were iteratively incorporated into subsequent focus groups.

### Phase 8: A consensus meeting with clinical experts

Prior to the online consensus meeting, the clinical experts who had participated in the Delphi study received a blinded feedback report containing their individual scores and group-level statistics for each PROM and PREM. These statistics included the percentage of responses within score ranges 1–3, 4–5, and 6–7, as well as the minimum, median, mean, interquartile range (IQR), and maximum scores. The report also included the recommendations from the methodologists and the results of the patient focus groups.

### Phase 9: Finalization by the research group

The research group finalized the selection based on the findings from all preceding phases, including evidence from the literature, expert consensus, and patient input.

### Reflexivity

Researcher involvement was considered across all phases of the study. In Phase 1, title screening was conducted independently by two researchers (GL and CL), reducing individual interpretation bias. CL’s prior experience with PROMs and PREMs in stroke research contributed to methodological expertise. Only instruments available in English and Dutch were included, which may have led to the exclusion of relevant measures in other languages. Subsequent phases were primarily conducted by a single researcher (GL), with predefined criteria applied where possible. Key decisions (Phase 4 and 9) were made in collaboration with the full research team. Additional perspectives were incorporated through input from two independent methodological experts (Phase 6). To minimize researcher influence, focus groups were moderated by an external partner (Flemish Patients’ Platform).

## Results

### Phase 1: Literature-based identification of PROMs and PREMs

The scoping literature search yielded 1203 records from which 1026 were excluded after title screening. Of the remaining 177 articles, a total of 41 PROMs and 17 PREMs were identified. These are listed under literature in Tables 1, 2, 3 and 4.

**Table 1:** Phase-by-phase summary of PROM additions and removals – Lung cancer

	Literature (Phase 1)	Hospital input (Phase 2)	Preliminary screening (Phase 4)	Modified Delphi* (Phase 5)	Methodologists interview (Phase 6)	Patient Focus Groups (Phase 7)	Consensus meeting (Phase 8)	Finalization by the research group (Phase 9)
Generic	• SF-36 • EQ-5D-5L • HADS • GAD-7 • PHQ-9 • PROMIS • SF-12 • Katz ADL scale • Rosow-Breslau IADL-scale • Nagi Disability scale • Spitzer Uniscale	**ADDED** • MMAS-8 • Welzijnsmeter	**REMOVED** • Rosow-Breslau IADL-scale • Nagi Disability scale • Spitzer Uniscale • MMAS-8 • Welzijnsmeter	• SF-36 (-) • EQ-5D-5L (-) • HADS (-) • GAD-7 (-) • PHQ-9 (-) • PROMIS (-) • SF-12 (-) • Katz ADL scale (-)	**ADDED** • WHODAS	• EQ-5D-5L • WHODAS	• EQ-5D-5L	• EQ-5D-5L • SF-HLQ (9 items)
Cancer-specific	• EORTC QLQ-C30 • FACT-G • PRO-CTCAE • MDASI • Distress thermometer • ESAS • NA-ACP • POS • SCNS-LF59 • SCNS-SF31 • SPARC • FLIC • SDS	**ADDED** • /	**REMOVED** • NA-ACP • POS • SCNS-LF59 • SCNS-SF31 • SPARC	• EORTC QLQ-C30 (+) • FACT-G (-) • PRO-CTCAE (-) • MDASI (-) • Distress thermometer (-) • ESAS (-) • FLIC (-) • SDS (-)	**ADDED** • /	• EORTC QLQ-C30	• EORTC QLQ-C30 • PRO-CTCAE (adapted)	• EORTC QLQ-C30 • PRO-CTCAE (adapted)
Lung cancer	• EORTC QLQ-LC13 • EORTC QLQ-LC29 • FACT-L • LCSS • MDASI-LC • LCSI • CC-LC • NA-ALCP • PSL • NSCLC-SAQ	**ADDED** • MRC Dyspnoea scale	**REMOVED** • LCSI • CC-LC • NA-ALCP • PSL	• EORTC QLQ-LC13 (§) • EORTC QLQ-LC29 (§) • FACT-L (-) • LCSS (-) • MDASI-LC (-) • NSCLC-SAQ (-) • MRC Dyspnoea scale (-)	**ADDED** • /	• EORTC QLQ-LC29	• EORTC QLQ-LC29	• EORTC QLQ-LC29

Abbreviations: SF-36 = Short Form (36) Health Survey; EQ-5D-5L = European Quality of Life Questionnaire – 5 Dimensions – 5 Levels; HADS = Hospital Anxiety and Depression Scale; GAD-7 = General Anxiety Disorder – 7; PHQ-9 = Patient Health Questionnaire – 9; PROMIS = Patient-Reported Outcome Measurement Information System; SF-12 = Short Form (12) Health Survey; Katz ADL scale = Katz Activities of Daily Living Scale; Rosow Breslau IADL-scale = Rosow-Breslau Instrumental Activities of Daily Living scale; EORTC QLQ-C30 = European Organization for Research and Treatment of Cancer Quality of Life Questionnaire – Core Module 30; FACT-G = Functional Assessment of Cancer Therapy – General; PRO-CTCAE = Patient-Reported Outcomes version of the Common Terminology Criteria for Adverse Events; MDASI = MD Anderson Symptom Inventory; ESAS = Edmonton Symptom Assessment Scale; NA-ACP = Needs Assessment for Advanced Cancer Patients; POS = Palliative Outcome Scale; SCNS-LF59 = Supportive Care Needs Survey – Long Form 59; SCNS-SF31 = Supportive Care Needs Survey – Short Form 31; SPARC = Sheffield Profile for Assessment and Referral to Care; FLIC = Functional Living Index Cancer; SDS = Symptom Distress Scale; EORTC QLQ-CR29 = European Organization for Research and Treatment of Cancer Quality of Life Questionnaire – Colorectal Cancer 29; ABCRC-Tool = Assessment of Burden of ColoRectal Cancer tool; LARS = Low Anterior Resection Syndrome score; CCF-CaQL = Cleveland Clinic Colorectal Cancer Quality of Life Questionnaire; COREFO = Colorectal Functional Outcome; FACT-C = Functional Assessment of Cancer Therapy – Colorectal; MSKCC bowel function = Memorial Sloan Kettering Cancer Center bowel function; MMAS-8 = Morisky Medication Adherence Scale – 8; WHODAS = World Health Organization Disability Assessment Schedule

*Three symbols (-, +, and §) were used to indicate the proportion of respondents assigning an overall score of 6 or 7 to each questionnaire: (-) <50% of respondents, (§) 50-74% of respondents, and (+) ≥75% of respondents

**Table 2:** Phase-by-phase summary of PROM additions and removals – Rectal cancer

	Literature (Phase 1)	Hospital input (Phase 2)	Preliminary screening (Phase 4)	Modified Delphi* (Phase 5)	Methodologists interview (Phase 6)	Patient Focus Groups (Phase 7)	Consensus meeting (Phase 8)	Finalization by the research group (Phase 9)
Generic	• SF-36 • EQ-5D-5L • HADS • GAD-7 • PHQ-9 • PROMIS • SF-12 • Katz ADL scale • Rosow-Breslau IADL-scale • Nagi Disability scale • Spitzer Uniscale	**ADDED** • MMAS-8 • Welzijnsmeter	**REMOVED** • Rosow-Breslau IADL-scale • Nagi Disability scale • Spitzer Uniscale • MMAS-8 • Welzijnsmeter	• SF-36 (-) • EQ-5D-5L (§) • HADS (-) • GAD-7 (-) • PHQ-9 (-) • PROMIS (-) • SF-12 (-) • Katz ADL scale (-)	**ADDED** • WHODAS	• EQ-5D-5L • WHODAS	• EQ-5D-5L	• EQ-5D-5L • SF-HLQ (9 items)
Cancer-specific	• EORTC QLQ-C30 • FACT-G • PRO-CTCAE • MDASI • Distress thermometer • ESAS • NA-ACP • POS • SCNS-LF59 • SCNS-SF31 • SPARC • FLIC • SDS	**ADDED** • /	**REMOVED** • NA-ACP • POS • SCNS-LF59 • SCNS-SF31 • SPARC	• EORTC QLQ-C30 (§) • FACT-G (-) • PRO-CTCAE (-) • MDASI (-) • Distress thermometer (-) • ESAS (-) • FLIC (-) • SDS (-)	**ADDED** • /	• EORTC QLQ-C30	• EORTC QLQ-C30 • PRO-CTCAE (adapted)	• EORTC QLQ-C30 • PRO-CTCAE (adapted)
Rectal cancer	• EORTC QLQ-CR29 • ABCRC-Tool • LARS • CCF-CaQL • COREFO • FACT-C • MSKCC bowel function	**ADDED** • Wexner score	**REMOVED** • /	• EORTC QLQ-CR29 (§) • ABCRC-Tool (-) • LARS (+) • CCF-CaQL (-) • COREFO (-) • FACT-C (-) • MSKCC bowel function (-) • Wexner score (-)	**ADDED** • /	• EORTC QLQ-CR29 • LARS • COREFO	• EORTC QLQ-CR29 • LARS	• EORTC QLQ-CR29 • EORTC QLQ-LM21 (1 item) • LARS

Abbreviations: SF-36 = Short Form (36) Health Survey; EQ-5D-5L = European Quality of Life Questionnaire – 5 Dimensions – 5 Levels; HADS = Hospital Anxiety and Depression Scale; GAD-7 = General Anxiety Disorder – 7; PHQ-9 = Patient Health Questionnaire – 9; PROMIS = Patient-Reported Outcome Measurement Information System; SF-12 = Short Form (12) Health Survey; Katz ADL scale = Katz Activities of Daily Living Scale; Rosow Breslau IADL-scale = Rosow-Breslau Instrumental Activities of Daily Living scale; EORTC QLQ-C30 = European Organization for Research and Treatment of Cancer Quality of Life Questionnaire – Core Module 30; FACT-G = Functional Assessment of Cancer Therapy – General; PRO-CTCAE = Patient-Reported Outcomes version of the Common Terminology Criteria for Adverse Events; MDASI = MD Anderson Symptom Inventory; ESAS = Edmonton Symptom Assessment Scale; NA-ACP = Needs Assessment for Advanced Cancer Patients; POS = Palliative Outcome Scale; SCNS-LF59 = Supportive Care Needs Survey – Long Form 59; SCNS-SF31 = Supportive Care Needs Survey – Short Form 31; SPARC = Sheffield Profile for Assessment and Referral to Care; FLIC = Functional Living Index Cancer; SDS = Symptom Distress Scale; EORTC QLQ-CR29 = European Organization for Research and Treatment of Cancer Quality of Life Questionnaire – Colorectal Cancer 29; ABCRC-Tool = Assessment of Burden of ColoRectal Cancer tool; LARS = Low Anterior Resection Syndrome score; CCF-CaQL = Cleveland Clinic Colorectal Cancer Quality of Life Questionnaire; COREFO = Colorectal Functional Outcome; FACT-C = Functional Assessment of Cancer Therapy – Colorectal; MSKCC bowel function = Memorial Sloan Kettering Cancer Center bowel function; MMAS-8 = Morisky Medication Adherence Scale – 8; WHODAS = World Health Organization Disability Assessment Schedule

*Three symbols (-, +, and §) were used to indicate the proportion of respondents assigning an overall score of 6 or 7 to each questionnaire: (-) <50% of respondents, (§) 50-74% of respondents, and (+) ≥75% of respondents

**Table 3:** Phase-by-phase summary of PREM additions and removals – Lung cancer

	Literature (Phase 1)	Hospital input (Phase 2)	Preliminary screening (Phase 4)	Modified Delphi* (Phase 5)	Methodologists interview (Phase 6)	Patient Focus Groups (Phase 7)	Consensus meeting (Phase 8)	Finalization by the research group (Phase 9)
Generic	• FACIT-TS-G • FACIT-TS-PS • CAHPS • PainCQ • PPCSS • M-PICS	**ADDED** • /	**REMOVED** • PainCQ	• FACIT-TS-G (-) • FACIT-TS-PS (-) • CAHPS (-) • PPCSS (-) • M-PICS (-)	**ADDED** • OECD-items	• Vlaamse Patiënten Peiling • OECD-items	PREM needs to be developed	PREM needs to be developed
Cancer-specific	• CTSQ • EORTC IN-PATSAT32 • PSCC • APECC • CCCQ-P • CPIIST • MCQ • OPPQNCS • PASQOC • SCIP	**ADDED** • /	**REMOVED** • OPPQNCS • PASQOC	• CTSQ (-) • EORTC IN-PATSAT32 (-) • PSCC (-) • APECC (-) • CCCQ-P (-) • CPIIST (-) • MCQ (-) • SCIP (-)	**ADDED** • /	• EORTC IN-PATSAT32		
Lung cancer	• Indicators NSCLC	**ADDED** • /	**REMOVED** • /	• Indicators NSCLC (-)	**ADDED** • /	/		

Abbreviations: FACIT-TS-G = Functional Assessment of Chronic Illness Therapy – Treatment Satisfaction – General; FACIT-TS-PS = Functional Assessment of Chronic Illness Therapy – Treatment Satisfaction – Patient Satisfaction; CAHPS = Consumer Assessment of Healthcare Providers and Systems; PainCQ = Pain Care Quality Survey; PPCSS = Perceived Physician's Communication Style Scale; M-PICS = Modified – Perceived Involvement in Care Scale; CTSQ = Cancer Therapy Satisfaction Questionnaire; EORTC IN-PATSAT32 = European Organization for Research and Treatment of Cancer Inpatient Satisfaction with Care Questionnaire 32; PSCC = Patient Satisfaction with Cancer-related Care; APECC = Assessment of Patient Experiences of Cancer Care; CCCQ-P = Cancer Care Coordination Questionnaire for Patients; CPIIST = Cancer Patient Information Importance and Satisfaction Tool; MCQ = Medical Care Questionnaire; OPPQNCS = Oncology Patients' Perceptions of the Quality of Nursing Care Scale; PASQOC = Patient Satisfaction and Quality Of life in Cancer outpatients; SCIP = Satisfaction with Cancer Information Profile; Indicators NSCLC = Indicators Non-Small Cell Lung Cancer; OECD items = Organisation for Economic Co-operation and Development items

*Three symbols (-, +, and §) were used to indicate the proportion of respondents assigning an overall score of 6 or 7 to each questionnaire: (-) <50% of respondents, (§) 50-74% of respondents, and (+) ≥75% of respondents

**Table 4:** Phase-by-phase summary of PREM additions and removals – Rectal cancer

	Literature (Phase 1)	Hospital input (Phase 2)	Preliminary screening (Phase 4)	Modified Delphi* (Phase 5)	Methodologists interview (Phase 6)	Patient Focus Groups (Phase 7)	Consensus meeting (Phase 8)	Finalization by the research group (Phase 9)
**Generic**	• FACIT-TS-G • FACIT-TS-PS • CAHPS • PainCQ • PPCSS • M-PICS	**ADDED** • /	**REMOVED** • PainCQ	• FACIT-TS-G (-) • FACIT-TS-PS (-) • CAHPS (-) • PPCSS (-) • M-PICS (-)	**ADDED** • OECD-items	• Vlaamse Patiënten Peiling • OECD-items	PREM needs to be developed	PREM needs to be developed
**Cancer-specific**	• CTSQ • EORTC IN-PATSAT32 • PSCC • APECC • CCCQ-P • CPIIST • MCQ • OPPQNCS • PASQOC • SCIP	**ADDED** • /	**REMOVED** • OPPQNCS • PASQOC	• CTSQ (-) • EORTC IN-PATSAT32 (-) • PSCC (-) • APECC (-) • CCCQ-P (-) • CPIIST (-) • MCQ (-) • SCIP (-)	**ADDED** • /	• EORTC IN-PATSAT32		
**Rectal cancer**	/	**ADDED** • /	**REMOVED** • /	/	**ADDED** • /	/		

Abbreviations: FACIT-TS-G = Functional Assessment of Chronic Illness Therapy – Treatment Satisfaction – General; FACIT-TS-PS = Functional Assessment of Chronic Illness Therapy – Treatment Satisfaction – Patient Satisfaction; CAHPS = Consumer Assessment of Healthcare Providers and Systems; PainCQ = Pain Care Quality Survey; PPCSS = Perceived Physician's Communication Style Scale; M-PICS = Modified – Perceived Involvement in Care Scale; CTSQ = Cancer Therapy Satisfaction Questionnaire; EORTC IN-PATSAT32 = European Organization for Research and Treatment of Cancer Inpatient Satisfaction with Care Questionnaire 32; PSCC = Patient Satisfaction with Cancer-related Care; APECC = Assessment of Patient Experiences of Cancer Care; CCCQ-P = Cancer Care Coordination Questionnaire for Patients; CPIIST = Cancer Patient Information Importance and Satisfaction Tool; MCQ = Medical Care Questionnaire; OPPQNCS = Oncology Patients' Perceptions of the Quality of Nursing Care Scale; PASQOC = Patient Satisfaction and Quality Of life in Cancer outpatients; SCIP = Satisfaction with Cancer Information Profile; Indicators NSCLC = Indicators Non-Small Cell Lung Cancer; OECD items = Organisation for Economic Co-operation and Development items

*Three symbols (-, +, and §) were used to indicate the proportion of respondents assigning an overall score of 6 or 7 to each questionnaire: (-) <50% of respondents, (§) 50-74% of respondents, and (+) ≥75% of respondents

The PROMs included 11 generic, 13 cancer-specific, 10 lung cancer-specific, and seven rectal cancer-specific instruments. Generic PROMs included the Short Form (36) Health Survey (SF-36), European Quality of Life Questionnaire – 5 Dimensions – 5 Levels (EQ-5D-5L), Hospital Anxiety and Depression Scale (HADS), General Anxiety Disorder – 7 (GAD-7), Patient Health Questionnaire – 9 (PHQ-9), Patient-Reported Outcome Measurement Information System (PROMIS), Short Form (12) Health Survey (SF-12), Katz Activities of Daily Living Scale (Katz ADL scale), Rosow-Breslau Instrumental Activities of Daily Living – scale (Rosow Breslau IADL-scale), Nagi Disability scale and Spitzer Uniscale.

Cancer-specific PROMs included the European Organization for Research and Treatment of Cancer Quality of Life Questionnaire – Core Module 30 (EORTC QLQ-C30), Functional Assessment of Cancer Therapy – General (FACT-G), Patient-Reported Outcomes version of the Common Terminology Criteria for Adverse Events (PRO-CTCAE), MD Anderson Symptom Inventory (MDASI), Distress thermometer, Edmonton Symptom Assessment Scale (ESAS), Needs Assessment for Advanced Cancer Patients (NA-ACP), Palliative Outcome Scale (POS), Supportive Care Needs Survey – Long Form 59 (SCNS-LF59), Supportive Care Needs Survey – Short Form 31 (SCNS-SF31) and Sheffield Profile for Assessment and Referral to Care (SPARC), Functional Living Index Cancer (FLIC) and Symptom Distress Scale (SDS).

Lung cancer-specific PROMs included the European Organization for Research and Treatment of Cancer Quality of Life Questionnaire – Lung Cancer 13 (EORTC QLQ-LC13), European Organization for Research and Treatment of Cancer Quality of Life Questionnaire – Lung Cancer 29 (EORTC QLQ-LC29), Functional Assessment of Cancer Therapy – Lung (FACT-L), Lung Cancer Symptom Scale (LCSS), MD Anderson Symptom Inventory – Lung Cancer (MDASI-LC), Lung Cancer Stigma Inventory (LCSI), Concerns Checklist-Lung Cancer (CC-LC), Needs Assessment for Advanced Lung Cancer Patients (NA-ALCP), Problem Symptom List (adapted form Distress Management Tool) (PSL) and Non-Small Cell Lung Cancer Symptom Assessment Questionnaire (NSCLC-SAQ).

Rectal cancer-specific PROMs included the European Organization for Research and Treatment of Cancer Quality of Life Questionnaire – Colorectal Cancer 29 (EORTC QLQ-CR29), Assessment of Burden of ColoRectal Cancer - tool (ABCRC-Tool), Low Anterior Resection Syndrome score (LARS), Cleveland Clinic Colorectal Cancer Quality of Life Questionnaire (CCF-CaQL), Colorectal Functional Outcome (COREFO), Functional Assessment of Cancer Therapy - Colorectal (FACT-C) and Memorial Sloan Kettering Cancer Center bowel function (MSKCC bowel function).

In addition, 17 PREMs were identified, including six generic, 10 cancer-specific, and one lung cancer-specific PREM. The generic PREMs included the Functional Assessment of Chronic Illness Therapy – Treatment Satisfaction – General (FACIT-TS-G), Functional Assessment of Chronic Illness Therapy – Treatment Satisfaction – Patient Satisfaction (FACIT-TS-PS), Consumer Assessment of Healthcare Providers and Systems (CAHPS), Pain Care Quality Survey (PainCQ), Perceived Physician’s Communication Style Scale (PPCSS) and Modified – Perceived Involvement in Care Scale (M-PICS). Cancer-specific PREMs included the Cancer Therapy Satisfaction Questionnaire (CTSQ), European Organization for Research and Treatment of Cancer Inpatient Satisfaction with Care Questionnaire 32 (EORTC IN-PATSAT32), Patient Satisfaction with Cancer-related Care (PSCC), Assessment of Patient Experiences of Cancer Care (APECC), Cancer Care Coordination Questionnaire for Patients (CCCQ-P), Cancer Patient Information Importance and Satisfaction Tool (CPIIST), Medical Care Questionnaire (MCQ), Oncology Patients’ Perceptions of the Quality of Nursing Care Scale (OPPQNCS), Patient Satisfaction and Quality Of life in Cancer outpatients (PASQOC) and Satisfaction with Cancer Information Profile (SCIP). One lung cancer-specific PREM was identified: Indicators Non-Small Cell Lung Cancer (Indicators NSCLC). No rectal cancer-specific PREMs were found.

### Phase 2: Inventory of PROMs and PREMs currently used and proposed by the participating hospitals

Eight of the 10 participating hospitals contributed to this phase by providing an overview of the PROMs and PREMs currently used in lung and rectal cancer care and by suggesting additional relevant instruments for inclusion in the selection process. Two generic PROMs (Morisky Medication Adherence Scale-8 or MMAS-8 and Welzijnsmeter), one lung cancer-specific PROM (Medical Research Council Dyspnoea scale or MRC Dyspnoea Scale) and one rectal cancer-specific PROM (Wexner Score) were added to the further selection process. An overview of the currently used and proposed measures is provided in Table 5; the added instruments are also reflected in Tables 1, 2, 3 and 4.

**Table 5:** Inventory of PROMs and PREMs currently used and proposed by the participating hospitals

	Currently used measures for lung cancer	Currently used measures for rectal cancer	Suggested measures
Hospital 1	/	• Distress Thermometer • EQ-5D • LARS • EORTC QLQ-CR29 • EORTC QLQ-C30	/
Hospital 2	/	• EQ-5D • LARS	Lung cancer: • ICHOM questionnaires Rectal cancer: • COREFO • Female sexual function index (FSFI) • Female sexual distress scale – revised (FSDS-R) • International Index of Erectile Function (IIEF-5) • International Prostate Symptom Score (IPSS)
Hospital 3	Welzijnsmeter	Welzijnsmeter	/
Hospital 4	• EQ-5D • PRO CTCAE • EORTC QLQ-C30 • EORTC QLQ-LC29	/	/
Hospital 5	• PRO-CTCAE	/	• PRO-CTCAE • EORTC QLQ-C30 • EORTC QLQ-LC29 • EORTC QLQ-CR29
Hospital 6	• EQ-5D • PRO-CTCAE • MRC Dyspnea Scale • MMAS-8	• LARS • Wexnerscore	Lung cancer: • Distress thermometer • EORTC QLQ IN-PATSAT32, • EORTC QLQ-LC29 • EORTC QLQ-LC13 • LCSS Rectal cancer: • Distress thermometer • Return-to-work outcomes • Outcomes and experiences related to intimacy and sexuality • Polyneuropathy related to neoadjuvant oxaliplatin-based chemotherapy
Hospital 7	• SF-36 • EQ-5D • EORTC QLQ-LC13 • EORTC QLQ-C30 • PHQ-9	/	/
Hospital 8	• EORTC QLQ-C30 • EORTC QLQ-LC29	• PRO-CTCAE • LARS • Wexnerscore	/

### Phase 3 & 4: Systematic assessment of PROM and PREM characteristics and preliminary selection by the research team

After an in-depth search, the research group evaluated information on nine predefined characteristics. PROMs and PREMs for which information was unavailable for four or more of these characteristics were excluded. A total of 17 instruments were removed during the preliminary screening phase. The following generic PROMs were excluded: Rosow Breslau IADL-scale, Nagi Disability scale and Welzijnsmeter. The following cancer-specific PROMs were excluded: NA-ACP, POS, SCNS-LF59, SCNS-SF31 and SPARC. Three lung cancer-specific PROMs were excluded: CC-LC, NA-ALCP, and PSL, as well as one generic PREM: Pain CQ, and two cancer-specific PREMs: OPPQNCS and PASQOC. Most excluded instruments lacked information regarding normative data, costs, available languages, and prognostic value. An overview of the excluded instruments and the corresponding missing information is provided in Table 6.

**Table 6:** PROMs and PREMs excluded during preliminary screening due to insufficient available information

Category	Instrument	Missing information leading to exclusion
Generic PROMs	Rosow Breslau IADL-scale	Normative data, costs, available languages, reliability, prognostic value
	Nagi Disability Scale	Response categories, normative data, costs, available languages, validity, prognostic value
	Welzijnsmeter	No information available
Cancer-specific PROMs	NA-ACP	Normative data, costs, available languages, validity*, reliability*, prognostic value
	POS	Normative data, costs, available languages, validity*, reliability*, prognostic value
	SCNS-LF59	Normative data, costs, available languages, validity*, reliability*, prognostic value
	SCNS-SF31	Normative data, costs, available languages, validity*, reliability*, prognostic value
	SPARC	Normative data, costs, available languages, validity*, reliability*, prognostic value
Lung cancer-specific PROMs	CC-LC	Normative data, costs, available languages, validity, reliability, prognostic value
	NA-ALCP	Normative data, costs, available languages, prognostic value
	PSL	Normative data, costs, available languages, validity, reliability, prognostic value
Generic PREMs	PainCQ	Normative data, costs, available languages, prognostic value
Cancer-specific PREMs	OPPQNCS	Normative data, costs, available languages, prognostic value
	PASQOC	Normative data, costs, available languages, prognostic value

*Validity and reliability information was only available from the developer

In addition, three PROMs were excluded based on content considerations. The Spitzer Uniscale (generic PROM) was excluded as it consists of a single item, limiting its comprehensiveness. The MMAS-8 (generic PROM) was excluded as it assess medication adherence rather than patient-reported health outcomes. The LCSI (lung cancer-specific PROM) was excluded as it focuses on stigma, which was considered outside the scope of relevant patient-reported outcomes for this study. The excluded instruments are reflected under preliminary screening in Tables 1, 2, 3 and 4.

### Phase 5: A modified Delphi study with clinical experts

In total, 26 experts participated in the modified Delphi study. The lung cancer panel consisted of 13 experts, including 9 oncologists and 4 nurse practitioners from across the 10 hospitals, with no more than 2 participants from a single hospital. The rectal cancer panel also included 13 experts: 6 oncologists, 3 surgeons and 4 nurse practitioners from 8 of the 10 hospitals; 4 experts originated from the same hospital (1 oncologist, 2 surgeons, and 1 nurse); however, this did not affect the overall conclusions of the Delphi study. All instruments were evaluated using a 7-point Likert scale. Both expert groups assessed the same generic and cancer-specific PROMs and PREMs. In addition, lung cancer experts evaluated lung cancer specific measurements, whereas rectal cancer experts evaluated rectal cancer-specific measurements. In total, 31 PROMs were evaluated, including eight generic, eight cancer-specific, seven lung cancer-specific and eight rectal cancer-specific PROMs. Furthermore, 14 PREMs were evaluated, including five generic PREMs, eight cancer-specific PREMs, one lung cancer-specific PREM; no rectal cancer-specific PREMs were identified. The evaluated instruments are listed under modified Delphi in Tables 1, 2, 3 and 4.

Only the overall scores were included in the analysis, as the domain-specific ratings (validity, importance, feasibility, and usefulness) were optional and had low completion rates, with fewer than half of the experts completing these items.

Only the EORTC QLQ-C30 (lung cancer Delphi) and LARS (rectal cancer Delphi) met the predefined threshold, with ≥ 75% of experts assigning an overall score of 6 or 7. The EORTC QLQ-LC13, EORTC QLQ-LC29 (lung cancer Delphi) and EORTC QLQ-CR29 (rectal cancer Delphi) and the EQ-5D-5L (rectal cancer Delphi) achieved intermediate scores with 50–74% of experts assigning a score of 6 or 7. All other PROMs received scores below this threshold, with fewer than 50% of experts assigning a score of 6 or 7. None of the PREMs reached the predefined threshold.

As the overall Delphi scores were relatively low and additional instruments were considered necessary for further evaluation, the research team decided to also include instruments with a minimum agreement level of 50%. Consequently, the EQ-5D-5L, EORTC QLQ-LC29, and EORTC QLQ-CR29 were retained for further discussion. The EORTC QLQ-LC29 was selected over the EORTC QLQ-LC13 because both instruments achieved identical Delphi scores, while the EORTC QLQ-LC29 represents the more recent and comprehensive version. A detailed overview of the questionnaire scores is provided in Appendix 1.

### Phase 6: An interview with methodologists

Two methodologists reviewed the selection process and the preliminary set of instruments. They considered the overall methodological approach to be appropriate and well-structured but noted that the selected questionnaires were predominantly focused on clinical outcomes and lacked coverage of broader, societal aspects such as participation and social functioning (e.g. work, social activities, and relationships). To address this gap and to better support the value-based use of PROMs and PREMs for healthcare evaluation and quality improvement, they recommended the inclusion of the World Health Organization Disability Assessment Schedule (WHODAS) as an additional PROM and OECD-based items as a PREM. These additions are reflected under methodologists interview in Tables 1, 2, 3 and 4.

### Phase 7: Patient focus groups

The EQ-5D-5L, EORTC QLQ-C30, EORTC QLQ-LC29, EORTC QLQ-CR29, LARS (based on the Modified Delphi scores), WHODAS (following the methodologists’ recommendations) and COREFO (at clinicians’ request) were discussed in the patient focus groups. As no PREM reached the predefined thresholds in the modified Delphi study, the research team selected two validated instruments (Vlaamse Patiëntenpeiling and EORTC IN-PATSAT32), supplemented with OECD-based items (following the methodologists’ recommendations). These are listed under patient focus groups in Tables 1, 2, 3 and 4.

In total, input was collected from 10 participants across three focus groups: four patients with lung cancer (all female), three patients with rectal cancer (all male), one male expert by experience (i.e. former lung cancer patient), and two experts by experience with rectal cancer (one male and one female). Participants raised several general considerations regarding PROMs and PREMs, including the following: all participants expressed willingness to complete lengthy questionnaires if the results would contribute to improved treatment and follow-up. They emphasized the importance of adequate recall periods and highlighted the relevance of domains related to mental health, work, memory, fatigue, taste, anxiety, anger, and treatment-related symptoms such as rashes or itching. Participants noted that repeated questions about end-of-life could be experienced as distressing; however, the provided questionnaires were generally not perceived as confrontational. Participants preferred questionnaires with both numerical and verbal scoring options. They dislike questions about aspects that do not typically change over time, such as education, which are unnecessary and time-consuming. Questionnaires should always be in Dutch, with other language options, and allow leaving questions unanswered or marked as ‘not applicable’. Feedback was mixed, but overall the EQ-5D-5L, EORTC QLQ-C30, EORTC QLQ-LC29, and EORTC QLQ-CR29 were considered the most suitable PROMs. No clear preference emerged for a PREM. A detailed overview of the feedback per instrument, including strengths and limitations, is provided in Appendix 2.

### Phase 8: A consensus meeting with clinical experts

Each clinical expert received a blinded feedback report, including their individual scores alongside measures of central tendency, recommendations from the methodologists and the patient focus group results. These findings were subsequently discussed during an online consensus meeting, where all experts unanimously agreed on the proposed selection. The resulting PROM set included the EQ-5D-5L, EORTC QLQ-C30, EORTC QLQ-LC29, EORTC QLQ-CR29 and LARS. In addition, an adapted version of the PRO-CTCAE was included following discussion with the clinical experts, who considered it particularly relevant for symptom monitoring and treatment-related adverse events. No PREM was selected. The selected instruments are reflected under consensus meeting in Tables 1, 2, 3 and 4.

### Phase 9: Finalization by the research group

The research team finalized the selection based on the findings from all preceding phases. One additional item from the European Organization for Research and Treatment of Cancer Quality of Life Questionnaire – Liver Metastases module (EORTC QLQ-LMC21) regarding tingling in the hands and feet was included in the final PROM set for rectal cancer patients. This symptom was identified by patients during the focus groups as a common treatment-related side effect but was not covered by the selected rectal cancer-specific instruments, whereas it was included in the lung cancer module (EORTC QLQ-LC29). In addition, nine work-related items from the Short Form Health and Labour Questionnaire (SF-HLQ) were included for both cancer types. These items were considered highly relevant by patients during the focus groups and were also recommended by the methodologists, while not being covered in the selected questionnaires.

The final PROM set includes the EQ-5D-5L (generic), EORTC QLQ-C30 (cancer-specific), adapted PRO-CTCAE (cancer-specific), EORTC QLQ-LC29 (lung cancer-specific), EORTC QLQ-CR29, LARS, and EORTC QLQ-LMC21 (1 item) (rectal cancer-specific), as well as nine items from the SF-HLQ.

## Discussion

This study applied a structured, multi-phase approach involving both patients and healthcare professionals to identify and select relevant PROMs and PREMs for lung and rectal cancer, with the aim of supporting standardized patient-reported outcome and experience measurement in Flemish hospitals. Lung and rectal cancer were selected because both cancer types are already included in the Flemish Institute for Quality of Care’s monitoring system, which systematically collects process and outcome indicators. The integration of PROMs and PREMs into this system represents an important step toward more patient-centered and value-based healthcare. This aligns with the growing body of literature emphasizing the importance of PROMs in oncology care, with evidence demonstrating improvements in symptom monitoring, quality of life, and even survival outcomes [23].

The final PROM set comprised a generic PROM (EQ-5D-5L), two cancer-specific PROMs (EORTC QLQ-C30 and adapted PRO-CTCAE), and disease-specific measures for lung cancer (EORTC QLQ-LC29) and rectal cancer (EORTC QLQ-CR29 and LARS). In addition, one item from the EORTC QLQ-LMC21 addressing tingling in the hands and feet and nine work-related items from the SF-HLQ were included. Each instrument was selected to capture specific domains of patient-reported outcomes. The EQ-5D-5L assesses overall health status and health-related quality of life, while the EORTC QLQ-C30 evaluates global quality of life, functioning (physical, emotional, and social), and common cancer-related symptoms. The adapted PRO-CTCAE captures symptomatic treatment-related adverse events from the patient perspective. Disease-specific modules further refine these assessments by addressing cancer-specific symptoms and functional outcomes. The additional item from the EORTC QLQ-LMC21 captures chemotherapy-induced peripheral neuropathy, whereas the SF-HLQ items extend the assessment to work ability, productivity, and broader societal participation.

The estimated completion time for the complete PROM set is approximately 30 min, depending on the disease-specific module administered and the patient’s condition. In total, the PROM set comprises 102 items for lung cancer patients and 108 items for rectal cancer patients. Although the number of items is substantial, efforts were made to balance comprehensiveness with feasibility by minimizing respondent burden while ensuring adequate coverage of clinically and socially relevant outcome domains.

Overall, the selected instruments capture a broad range of concepts, including health status, quality of life, symptom burden, treatment-related side effects, and disease-specific functional outcomes. The inclusion of work-related items further broadens the scope to societal participation and longer-term consequences of cancer and its treatment. Importantly, the selected PROMs are widely used in oncology research and clinical practice and align with international initiatives such as the International Consortium for Health Outcomes Measurement (ICHOM), thereby supporting the consistency of the selected set with existing consensus-based outcome frameworks. The selected PROMs are intended for longitudinal use throughout the care trajectory, enabling systematic monitoring of symptoms and quality of life over time. At the individual level, this may support shared decision-making, improve patient–provider communication, and facilitate the identification of unmet needs. At the organizational and healthcare system level, aggregated PROM data may support quality improvement initiatives, benchmarking across hospitals, and healthcare evaluation. These applications are increasingly recognized as central components of value-based healthcare and learning health systems, although implementation challenges remain [24, 25].

This study has several strengths. First, it involved multiple hospitals across Flanders, including both university and general hospitals, which likely enhances the representativeness of the findings within the Flemish healthcare context. Second, the use of separate modified Delphi studies for lung cancer and rectal cancer resulted in largely consistent findings across both cancer types, suggesting alignment in the key concepts and outcome domains considered relevant. Third, the inclusion of clinical experts, specialized nurses, and patients through focus groups ensured that both professional and patient perspectives were incorporated into the selection process. This likely enhances the relevance, acceptability and potential implementation of the selected PROMs in clinical practice [26].

Several limitations should also be considered. First, the literature search was limited to Medline, which may have restricted the scope of identified instruments, although this database covers most biomedical literature. Second, only PROMs and PREMs available in English and Dutch were included, potentially excluding relevant instruments developed in other languages. Third, the expert panel was relatively small and restricted to the Flemish healthcare context, without involvement of international experts. In addition, due to time and budget constraints, access to certain questionnaires and validated translations was limited. Several questionnaires were not directly accessible online and could only be obtained upon request from the original developers (e.g. FLIC, ABCRC tool, CCF-SQL, LCSS, PPCSS, M-PICS, CTSQ, PSCC, APECC and CPIIST). As described in the Methods section, Delphi experts primarily evaluated standardized summaries of predefined instrument characteristics rather than systematically reviewing the full questionnaire texts themselves. However, if experts wished to consult the full questionnaire text, they could either retrieve it independently or request it from the researcher (GL), after which it was provided when available. Finally, although the predefined 75% agreement threshold ensured a high level of consensus, it proved to be relatively stringent, resulting in only a limited number of instruments meeting this criterion. Therefore, the agreement threshold for inclusion was lowered to 50%.

Future research should focus on the implementation and evaluation of the selected PROM set in routine clinical practice, including its feasibility, acceptability, and impact on care processes and patient outcomes. As no suitable PREM was identified, further work is needed to develop and validate a context-appropriate PREM that adequately captures patient experiences across the cancer care trajectory. Furthermore, the methodology applied in this study may be transferable to other cancer types and could support broader standardization of patient-reported measures within the Flemish healthcare system.

## Conclusion

This study identified standardized PROM sets for patients with lung and rectal cancer using a structured multi-phase approach involving aliterature review, clinical experts, methodologists and patients. The final PROM set included the EQ-5D-5L (generic), EORTC QLQ-C30 and adapted PRO-CTCAE (cancer-specific), EORTC QLQ-LC29 (lung cancer-specific), and EORTC QLQ-CR29, EORTC QLQ-LMC21 (1 item), and LARS (rectal cancer-specific), supplemented with work-related items from the SF-HLQ. No PREM met the predefined selection criteria, highlighting the need for the development of a context-specific PREM. The selected PROMs may support standardized and longitudinal patient-reported outcome measurement across Flemish hospitals, facilitating quality improvement, benchmarking, and value-based healthcare initiatives.

## Supplementary Information

Below is the link to the electronic supplementary material.

Supplementary Material 1

## Data Availability

The datasets used and/or analysed during the current study are available from the corresponding author on reasonable request.
