# Supplementary material for: Selection of patient-reported outcome measures (PROMs) and patient-reported experience measures (PREMs) for long-term follow up of patients diagnosed with lung or rectal cancer
Source: J Patient Rep Outcomes. 2026 Jul 8;10:179. doi: 10.1186/s41687-026-01133-x (PMC13633215; doi:10.1186/s41687-026-01133-x)
Supplement: Supplementary file 1 [file 41687_2026_1133_MOESM1_ESM.pdf]

APPENDIX 1: DELPHI STUDY RESULTS

Appendix 1 shows the results of two Delphi studies, one for lung cancer and one for rectal cancer, each including 13 participants. Participants were asked to rate each questionnaire using a 7-point Likert scale. The results are presented across separate tables for generic, cancer-specific, and lung/rectal cancer-specific PROMs and PREMs. The tables present the percentage of participants assigning scores of 1–3, 4–5, and 6–7. The color coding is based on the proportion of participants assigning a score of 6 or 7, with green indicating ≥75%, yellow 50–74%, and red <50%. For each questionnaire, the minimum, median, mean, interquartile range (IQR), and maximum scores are also reported.

1. Lung Cancer

1.1 Generic PROMs

PROM	% score 1-3	% score 4-5	% score 6-7	Minimum	Median	Mean	IQR	Maximum
EQ-5D-5L	23	38,5	38,5	1	5	4,6	2	7
HADS	46	23	31	1	4	4	4	7
PHQ-9	46	23	31	1	4	4	4	7
Katz ADL scale	46	23	31	1	4	4	4	7
SF-12	31	46	23	2	5	4,3	2	6
GAD-7	46	31	23	1	4	3,7	3	7
PROMIS	31	53,5	15,5	2	4	4,2	2	7
SF-36	46	38,5	15,5	2	3,5	3,7	2	6

1.2 Generic PREMs

PREM	% score 1-3	% score 4-5	% score 6-7	Minimum	Median	Mean	IQR	Maximum
FACIT TS-G	54	23	23	1	3	3,7	3	6
M-PICS	61,5	23	15,5	1	3	3,2	2	7
FACIT TS-PS	69	15,5	15,5	1	3	3,1	2	6
PPCSS	61,5	30,5	8	1	3	3,1	2	7
CAHPS	46	54	0	1	4	3,3	2	5

1.3 Cancer-specific PROMs

PROM	% score 1-3	% score 4-5	% score 6-7	Minimum	Median	Mean	IQR	Maximum
EORTC QLQ-C30	0	15,5	84,5	4	7	6,3	1	7
PRO-CTCAE	15,5	38,5	46	3	5	5,2	2	7
FACT-G	23	38,5	38,5	2	5	4,8	2	7
ESAS	38,5	46	15,5	1	4	3,9	2	6
FLIC	30,5	61,5	8	1	4	3,8	1	6
MDASI	38,5	54	7,5	1	4	3,8	2	6
Distress Thermometer	38,5	61,5	0	1	4	3,6	1	5
SDS	38,5	61,5	0	1	4	3,5	1	5

1.4 Cancer-specific PREMs

PREM	% score 1-3	% score 4-5	% score 6-7	Minimum	Median	Mean	IQR	Maximum
CTSQ	46	38,5	15,5	1	4	3,6	1	6
MCQ	38,5	53,5	8	1	4	3,7	1	6
APECC	53,5	38,5	8	1	3	3,3	1	6
PSCC	31	69	0	1	4	3,5	1	5
EORTC INPATSAT-32	38,5	61,5	0	1	4	3,5	1	5
CPIIST	38,5	61,5	0	1	4	3,5	1	5
SCIP	38,5	61,5	0	1	4	3,5	1	5
CCCQ-P	46	54	0	1	4	3,5	1	5

1.5 Lung Cancer-specific PROMs

PROM	% score 1-3	% score 4-5	% score 6-7	Minimum	Median	Mean	IQR	Maximum
EORTC QLQ-LC13	0	31	69	4	6	5,9	2	7
EORTC QLQ-LC29	8	23	69	2	6	5,8	2	7
LCSS	23	31	46	1	5	4,9	3	7
FACT-L	23	38,5	38,5	1	5	4,8	2	7
MDASI-LC	15,5	54	30,5	1	4	4,5	2	7
MRC Dyspnoea scale	31	46	23	1	4	3,9	2	6
NSCLC-SAQ	23	69	8	1	4	3,8	0	6

1.6 Lung Cancer-specific PREMs

PREM	% score 1-3	% score 4-5	% score 6-7	Minimum	Median	Mean	IQR	Maximum
Indicators NSCLC	31	69	0	1	4	3,5	1	4

2. Rectal cancer

2.1 Generic PROMs

PROM	% score 1-3	% score 4-5	% score 6-7	Minimum	Median	Mean	IQR	Maximum
EQ-5D-5L	15,5	31	53,5	3	6	5,3	3	7
SF-36	15,5	53,5	31	1	5	4,6	2	7
HADS	23	46	31	1	5	4,5	2	6
Katz ADL scale	54	15,5	30,5	2	3	3,8	4	6
SF-12	23	61,5	15,5	1	4	4,2	1	7
PROMIS	30,5	61,5	8	1	4	3,6	1	6
GAD-7	38,5	53,5	8	1	4	3,6	2	6
PHQ-9	54	46	0	1	3	3,3	1	5

2.2 Generic PREMs

PREM	% score 1-3	% score 4-5	% score 6-7	Minimum	Median	Mean	IQR	Maximum
FACIT TS-PS	23	38,5	38,5	1	5	4,5	2	7
FACIT TS-G	46	31	23	1	4	3,6	3	6
CAHPS	46	31	23	1	4	3,6	3	6
PPCSS	61,5	30,5	8	1	3	3,2	1	6
M-PICS	54	46	0	1	3	3,2	1	5

2.3 Cancer-specific PROMs

PROM	% score 1-3	% score 4-5	% score 6-7	Minimum	Median	Mean	IQR	Maximum
EORTC QLQ-C30	8	23	69	3	6	5,5	1	7
Distress Thermometer	23	38,5	38,5	1	5	4,5	2	7
FACT-G	30,5	39	30,5	1	5	4,2	3	6
PRO-CTCAE	30,5	39	30,5	1	4	4,2	3	7
SDS	15,5	69	15,5	1	4	4,2	1	6
ESAS	31	53,5	15,5	1	4	3,8	3	6
MDASI	46	46	8	1	4	3,5	2	7
FLIC	46	46	8	1	4	3,5	2	6

2.4 Cancer-specific PREMs

PREM	% score 1-3	% score 4-5	% score 6-7	Minimum	Median	Mean	IQR	Maximum
CCCQ-P	23	61,5	15,5	1	4	3,8	1	6
SCIP	23	61,5	15,5	1	4	3,8	0	6
PSCC	30,5	54	15,5	1	4	3,6	3	6
EORTC INPATSAT-32	38,5	46	15,5	1	4	3,6	2	6
MCQ	46	46	8	1	4	3	2	6
CPIIST	23	77	0	1	4	3,5	0	5
CTSQ	31	69	0	1	4	3,5	1	5
APECC	31	69	0	1	4	3,2	2	5

2.5 Rectal cancer-specific PROMs

PROM	% score 1-3	% score 4-5	% score 6-7	Minimum	Median	Mean	IQR	Maximum
LARS	8	15	77	1	6	5,8	1	7
EORTC QLQ-CR29	8	38,5	53,5	3	6	5,4	1	7
FACT-C	31	23	46	1	4	4,4	3	7
Wexner score	15,5	46	38,5	1	5	4,8	2	7
ABCRC-Tool	23	38,5	38,5	1	5	4,5	2	7
COREFO	30,5	30,5	39	1	4	4,2	3	7
CCF-CaQL	23	46	31	1	4	4,2	2	7
MSKCC bowel function	23	54	23	1	4	4,1	1	6

2.6 Rectal cancer-specific PREMs

PREM	% score 1-3	% score 4-5	% score 6-7	Minimum	Median	Mean	IQR	Maximum
/	-	-	-	-	-	-	-	-

APPENDIX 2: PATIENT FEEDBACK PER PROM/PREM (FOCUS GROUPS)

2.1 Patient feedback on PROMs

	Strengths	Limitations
**GENERIC**		
EQ-5D-5L	• *Brief questionnaire, resulting in low respondent burden*<br>• *Broad applicability*	• *Limited number of items may restrict comprehensiveness*<br>• *Insufficient specificity (too general)*<br>• *Reflects only a single time point ("today")*
WHODAS	• *Includes questions on work (return to work)*	• *The distinction between response options (e.g. somewhat vs. moderately) is unclear*<br>• *Issues with phrasing*<br>• *Items requiring recall of the number of days were perceived as difficult*
**CANCER-SPECIFIC**		
EORTC QLQ-C30	• *Comprehensive questionnaire covering key domains*<br>• *Perceived as the most complete instrument*<br>• *Combination of verbal and numerical response options*	• *First five items lack a reference period*<br>• *Distinction between "long" and "short" walking distances is unclear*
**LUNG CANCER-SPECIFIC**		
EORTC QLQ-LC29	• *Comprehensive questionnaire covering key domains*<br>• *Perceived as a high-quality instrument*<br>• *Combination of verbal and numerical response options*	• *Items could be better grouped (e.g. questions on coughing)*
**RECTAL CANCER-SPECIFIC**		
EORTC QLQ-CR29	• *Comprehensive questionnaire covering key domains*<br>• *Includes items addressing the patient as "a person"*<br>• *Can be used throughout the entire care trajectory*	• *Options to skip questions should be provided for sensitive topics (e.g. sexuality and intimacy)*
LARS		• *Considered too specific*<br>• *Perceived as too technical*<br>• *Not applicable to many patients*
COREFO		• *Perceived as too technical*<br>• *Considered too long and overly detailed (leading to low motivation to complete)*<br>• *Items not perceived as aligned with current clinical practice*

2.2 Patient feedback on PREMs

	Strengths	Limitations
Flemish Patient Survey	• *Considered as a good questionnaire*<br>• *Distinction between response options (e.g. "never", "sometimes", "usually", "always") is clear*	• *Educational level and financial situation were perceived as not relevant*<br>• *Repetitive questioning (e.g. multiple questions about oneself) perceived as unnecessary and time-consuming*<br>• *Lack of consistency in response options (e.g. mixing YES/NO with frequency scales)*<br>• *YES/NO response options perceived as overly simplistic*<br>• *Lack of clarity regarding who is referred to as "staff"*
EORTC IN-PATSAT32	• *Comprehensive questionnaire covering key domains*<br>• *Combination of verbal and numerical response options*<br>• *Clear distinction between physicians, nurses and services*	• *Items related to nurses and physicians are not comparable (e.g. regarding interpersonal aspects)*<br>• *Lengthy questionnaire*
OECD items	• *Includes a "no response" option*<br>• *Brief questionnaire, resulting in low respondent burden*<br>• *Broad applicability*	• *Lack of clarity regarding who is referred to as "healthcare provider"*<br>• *Limited number of items may restrict comprehensiveness*<br>• *Insufficient specificity (too general)*
